# Heller myotomy in patients with prior endoscopic interventions vs the treatment-naïve

**DOI:** 10.1007/s00464-025-11661-0

**Published:** 2025-04-15

**Authors:** Nethra Jain, John O. Barron, Andrew J. Toth, Monisha Sudarshan, Madhusudhan Sanaka, Sadhvika Ramji, Saurav Adhikari, Sudish C. Murthy, Eugene H. Blackstone, Siva Raja, the Cleveland Clinic Esophageal Research Group, Daniel P. Raymond, Prashanthi Thota, Scott L. Gabbard, Mark E. Baker

**Affiliations:** 1 Department of Thoracic and Cardiovascular Surgery, Heart, Vascular, and Thoracic Institute, Cleveland Clinic, 9500 Euclid Avenue / Desk J4-133, Cleveland, OH 44915 USA; 2 Department of Gastroenterology and Hepatology, Digestive Disease and Surgery Institute, Cleveland Clinic, 9500 Euclid Avenue / Desk J4-133, Cleveland, OH 44915 USA; 3 Department of Quantitative Health Sciences, Research Institute, Cleveland Clinic, 9500 Euclid Avenue / Desk J4-133, Cleveland, OH 44915 USA

**Keywords:** Esophageal achalasia, Heller myotomy, Pneumatic dilation, Botulinum toxin

## Abstract

**Background:**

Definitive palliation for achalasia is surgical myotomy; however, patients frequently undergo endoscopic treatments prior to myotomy. Surgeons may perceive myotomy to be more challenging after prior treatments, due to scarring and fusion of dissection planes, but outcomes compared to the treatment-naïve remain unclear. Hence, we compared institutional Heller myotomy outcomes in patients who underwent pre-myotomy endoscopic treatments to those who did not.

**Methods:**

From 1/1/2010 to 1/1/2020, 436 patients underwent Heller myotomy for achalasia at Cleveland Clinic, 101 (23%) of whom had prior endoscopic intervention(s): 39 (39%) pneumatic dilation, 57 (56%) botulinum toxin injection, and 5 (4.9%) both (Prior group). Propensity score matching generated two groups of 101 pairs. Short-term outcomes and longitudinal postoperative symptom palliation (Eckardt score ≤ 3), esophageal emptying at five minutes, and reintervention were assessed and compared with the treatment-naïve (Naïve group).

**Results:**

There were no statistically significant differences in operative time, mucosal perforation, or length of stay between Prior and Naïve groups (*P* > .12). At 5 years, the probability of symptom palliation was 83% in the Prior Group vs 81% in the Naïve Group (*P* = .63) and complete esophageal emptying 23% vs 32% (*P* = .095). The cumulative number of reinterventions per 100 patients at 10 years was 7.9 in the Prior Group and 4.8 in the Naïve Group (*P* = .13).

**Conclusion:**

The perception of increased complexity of Heller myotomy in patients with prior endoscopic interventions does not translate to stastically significant differences in short- or long-term outcomes when compared to the treatment-naïve. A subtle longitudinal pattern of suboptimal esophageal emptying and increased reintervention for patients with prior intervention(s), suggests that, when possible, up-front myotomy may be preferred.

**Supplementary Information:**

The online version contains supplementary material available at 10.1007/s00464-025-11661-0.

Achalasia is an esophageal motility disorder characterized by absent peristalsis and insufficient relaxation of the lower esophageal sphincter [1]. Myotomy of the lower esophageal sphincter, achieved by either Heller myotomy or per-oral endoscopic myotomy (POEM), is the cornerstone of palliative therapy; however, patients often undergo other endoscopic treatments, such as pneumatic dilation and botulinum toxin injection, prior to myotomy [2]. Resultant scarring and fusion of tissue planes often leads to a more challenging surgical dissection [3, 4]; however, whether postoperative outcomes are affected is controversial [2–15]. Hence, we assessed procedural and longitudinal outcomes in patients with endoscopic Intervention(s) prior to Heller myotomy compared to patients who underwent Heller myotomy as their initial therapy.

## Patients and methods

### Patients

From 1/1/2010 to 1/1/2020, 436 patients with achalasia underwent Heller Myotomy at Cleveland Clinic, 101 (23%) of whom had previous endoscopic intervention(s): 39 (39%) prior pneumatic dilation (≥ 30 mm), 57 (56%) botulinum toxin injection, and 5 (4.9%) both (Supplemental Table 1). The percentage of all patients undergoing Heller myotomy who had prior intervention(s) remained stable throughout the study period (Supplemental Fig. 1). Of the 436 Heller myotomies, 428 (98%) included a Dor fundoplication, with 270 (62%) performed laparoscopically, 160 (37%) robotically, and 5 (1.1%) open (Supplemental Table 2).

**Table 1:** Demographic and preoperative clinical characteristics of patients after propensity matching

	Prior treatment (N = 101)		Treatment-naïve (N = 101)		
Variable	Available N	Count(%) or Mean ± SD	Available N	Count(%) or Mean ± SD	SMD^c^
Age	101	52 ± 14	101	53 ± 16	−6.3
Female sex	101	53 (52)	101	57 (56)	−8.0
Race	99		99		9.2
White		93 (94)		95 (96)	
Black		3 (3)		2 (2)	
Other		3 (3)		2 (2)	
Body mass index	96	26 ± 5.5	99	26 ± 4.6	4.6
ASA^a^ class	95		97		−9.2
I		1 (1.1)		3 (3.1)	
II		27 (28)		19 (20)	
III		51 (54)		59 (61)	
IV		16 (17)		16 (16)	
Achalasia subtype	80		82		−10
Type 1		21(26)		18 (22)	
Type 2		53(66)		57 (70)	
Type 3		3(3.8)		4 (4.9)	
Other		3(3.8)		3 (3.7)	
Preop TBE^b^ sigmoid esophagus	96	21(22)	94	20 (21)	1.5
Preoperative Eckardt score					
Dysphagia	41		38		.62
Absent		1 (2.4)		1 (2.6)	
Occasional		4 (9.8)		4 (11)	
Daily		6 (15)		3 (7.9)	
Every meal		30 (73)		30 (79)	
Regurgitation	41		38		.87
Absent		6 (15)		4 (11)	
Occasional		9 (22)		13 (34)	
Daily		18 (44)		10 (26)	
Every meal		8 (20)		11 (29)	
Chest Pain	41		38		.38
Absent		14 (34)		16 (42)	
Occasional		15 (37)		14 (37)	
Daily		8 (20)		5 (13)	
Every meal		4 (9.8)		3 (7.9)	
Weight Loss	41		38		.32
Absent		17 (41)		13 (34)	
< 5 kg		7 (17)		4 (11)	
5–10 kg		9 (22)		11 (29)	
> 10 kg		8 (20)		10 (26)	
Total Eckardt Score	41		38		
0		1 (2.4)		0 (0)	
1		2 (4.9)		1 (2.6)	
2		0 (0)		0 (0)	
3		1 (2.4)		2 (5.3)	
4		2 (4.9)		5 (13)	
5		6 (15)		2 (5.3)	
6		8 (20)		10 (26)	
7		7 (17)		3 (7.9)	
8		4 (9.8)		3 (7.9)	
9		6 (15)		8 (21)	
10		3 (7.3)		3 (7.9)	
11		1 (2.4)		1 (2.6)	

^a^American Society of Anesthesiologists

^b^Timed Barium Esophagram

^c^Standardized Mean Difference

**Fig. 1:**
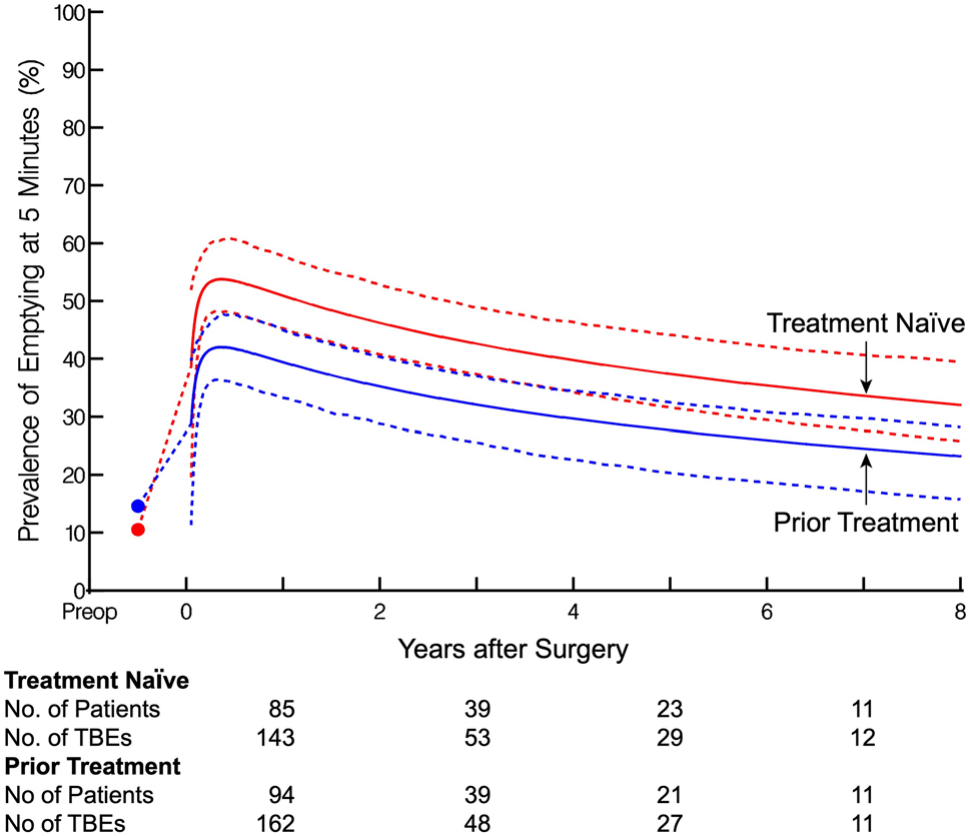
Adjusted longitudinal prevalence of complete TBE emptying at 5 min after Heller myotomy in patients with prior treatment (blue) vs the treatment-naïve (red). Solid lines represent the estimated model of complete emptying. Symbols represent actual data grouped over different time intervals without regard to the repeated observation, presented here as a crude verification of the model fit. The number of patients and TBEs available between are depicted below the graph (Color figure online)

**Table 2:** Perioperative and short-term outcomes after propensity matching

	Prior treatment (N = 101)		Treatment-naïve (N = 101)		
Variable	Available N	Count(%) or 15/50/85 percentile	Available N	Count(%) or 15/50/85 percentile	*P*-value
Surgical Approach	101		100		.7
Robotic		29 (29)		33 (33)	
Laparoscopic		70 (69)		64 (64)	
Open		2 (2.0)		3 (3.0)	
Fundoplication type	101		100		> .99
Dor		98 (97)		97 (97)	
No fundoplication		3 (3.0)		3 (3.0)	
Operative time, min	54	103/131/159	62	103/128/161	> .99
Intraoperative mucosal perforation	101	0 (0)	100	3 (3)	.12
Postoperative leak	101	1(0.99)	101	0(0)	> .99
Length of stay, days	101	1/1/2	101	1/1/2	.83
Symptoms^a^					
Dysphagia	84	36(43)	80	27(34)	.23
Regurgitation	84	15(18)	80	11(14)	.47
Chest Pain	81	9(11)	78	11(14)	.57
Total Eckardt Score^a^	45		46		.6
0		24 (53)		25 (54)	
1		16 (36)		11 (24)	
2		5 (11)		7 (15)	
3		0 (0)		1 (2.2)	
4		0 (0)		1 (2.2)	
5		0 (0)		1 (2.2)	
TBE^b^ Complete emptying					
1 min	91	14(15)	80	10 (13)	.59
5 min	92	40(43)	80	39 (49)	.49
DeMeester score	56		46		.18
Abnormal (> 14.72)		11(20)		6(13)	

^a^Measured within 6 months postoperatively

^b^Timed barium esophagram

### Patient evaluation

Patients were evaluated by a multidisciplinary team that included a gastroenterologist and thoracic surgeon. Preoperative evaluation included timed barium esophagram (TBE), high-resolution manometry, esophagogastroduodenoscopy (EGD), and symptom assessment with Eckardt score [16]. Before POEM availability in 2014, all patients were offered Heller myotomy, typically with Dor fundoplication. Since 2014, both POEM and Heller myotomy with Dor fundoplication were discussed with the patient, and final treatment decisions made as per our previously published algorithm [17].

### Surgical and postoperative management

Based on surgeon preference and era, Heller myotomy was performed either laparoscopically, robotically, or via the open approach, with increased use of a robotic approach since 2015. Myotomy and Dor fundoplication were performed as previously described by our group [18, 19]. A soluble contrast swallow radiograph was obtained on postoperative day 1, after which, if no evidence of leak, a liquid diet was started and gradually advanced over several days.

### Data and ethical considerations

Baseline, procedural, and postprocedural data were obtained from an Achalasia Registry prospectively maintained in a Research Electronic Data Capture (REDCap) database, medical record review, and follow-up for clinical surveillance. Cleveland Clinic’s Institutional Review Board approved use of all these data for research, with patient consent waived (#4826, with approval through December 2024).

### Endpoints

Endpoints were procedural outcomes, esophageal drainage assessed by TBE complete emptying (barium column height and width of 0 cm at 5 min), symptom palliation (expressed by Eckardt score ≤ 3), and esophageal reintervention (pneumatic dilation of ≥ 30 mm, POEM, repeat Heller myotomy, or esophagectomy) [20]. From 2010 to 2013, our method for postoperative symptom assessment documented the presence or absence of dysphagia, regurgitation, and chest pain. From 2014 onward, we transitioned to measurement of pre- and postoperative Eckardt scores. Early postoperative outcomes refer to TBE and symptoms within the first 6 postoperative months.

### Follow-up

Time zero was the date of Heller myotomy. Follow-up occurred at approximately 2 weeks, 3 months, and annually for 3 years via in-person visits or telephone calls. If patients had adequate symptom palliation and stable esophageal drainage at 3 years, they were monitored every 2 to 3 years thereafter. A pH study was recommended within the first 6 months. Postoperative manometry was not routinely performed. Eckardt score and TBE were recorded at every visit. EGD was performed at 5 years or as needed if symptoms or TBE emptying worsened. Median follow-up time for patients was 4.0 years; 25% of patients were followed > 7.8 years and 10% > 10.8 years (Supplemental Fig. 2).

**Fig. 2:**
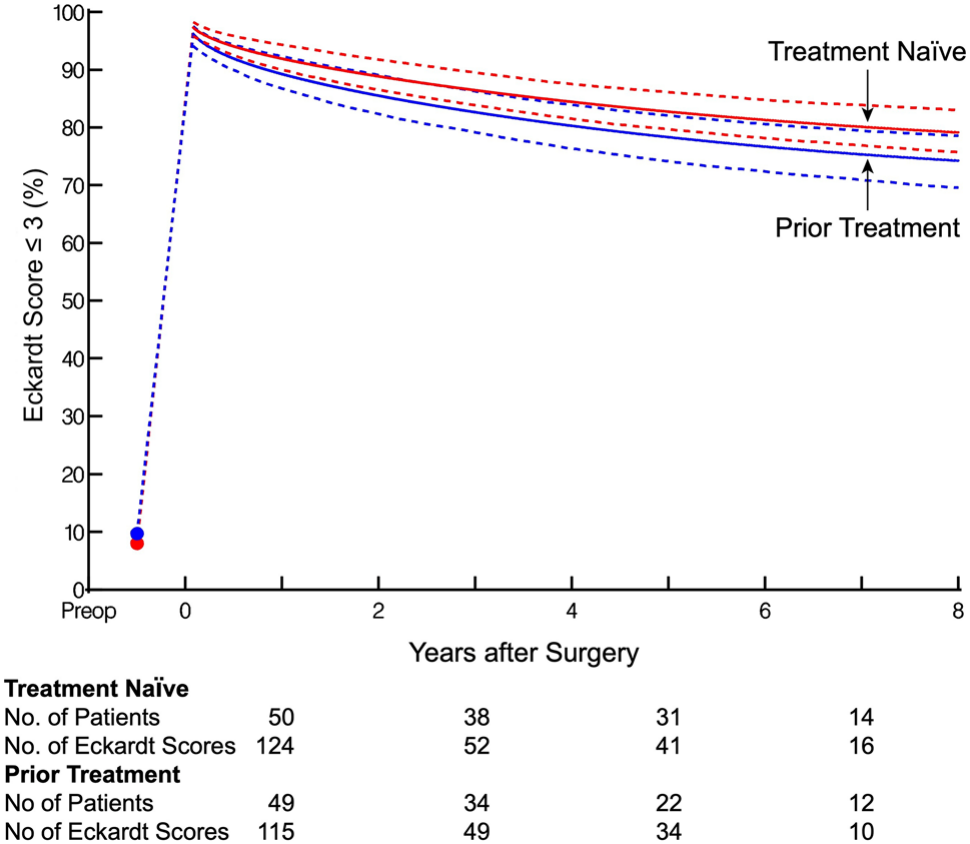
Adjusted longitudinal prevalence of Eckardt score ≤ 3 after Heller myotomy in prior treatment (blue) vs treatment-naïve (red) groups. Solid lines depict parametric estimates of prevalence of Eckardt scores. Symbols represent actual data grouped over different time intervals without regard to the repeated observation, presented here as a crude verification of the model fit. The number of patients and Eckardt scores available between time points are depicted below the graph (Color figure online)

### Statistical analysis

Analyses were performed with SAS v9.4 (SAS Institute Inc., Cary, NC, USA), and R v4.2.1 (R Foundation for Statistical Computing, https://www.r-project.org/) statistical software. Continuous variables are presented as mean ± standard deviation (SD), or median [15th, 85th percentiles]. Categorical variables are presented as frequencies and percentages. Comparisons were made using the Wilcoxon rank sum (Kruskal–Wallis) non-parametric test for continuous variables, and the χ^2^ test for categorical variables. Uncertainty is expressed by confidence limits (CL) equivalent to ± 1 standard error (68%).

#### Propensity matching

We compared outcomes of the groups by employing propensity score matching based on demographic and preoperative variables (Supplemental Table 3). We first imputed missing values using fivefold multiple imputation by chained equations (MICE, SAS PROC MI) [21]. All listed variables were incorporated into a saturated multivariable logistic regression model for each imputed dataset. For each imputed dataset, the probability of group membership was then calculated for each patient and the 5 probabilities averaged to produce a propensity score.

**Table 3:** Number and types of reinterventions in the matched cohort

	Prior Treatment (N = 101)	Treatment-naïve (N = 101)
Total	24	15
Pneumatic dilation	14	10
POEM^a^	7	3
Heller myotomy	0	1
Esophagectomy	3	1

^a^Per-Oral Endoscopic Myotomy

Using the propensity score, patients undergoing prior treatments were matched with treatment-naïve patients using 1:1 greedy matching [22]. The maximum allowable difference in propensity scores was set to 0.25 times the standard deviation of the logit of the propensity score (0.25 × SD[logit(propensity score)] = 0.16) [23]. Treatment-naïve patients whose propensity score deviated more than 0.1 were considered unmatched. This resulted in 101 pairs (100% of possible matches) with a C-statistic of 0.68. Standardized mean differences were used to assess the distribution of patient characteristics before and after matching (Supplemental Fig. 3), and mirrored histograms employed to demonstrate the distribution of propensity scores for matched and unmatched groups (Supplemental Fig. 4).

**Fig. 3:**
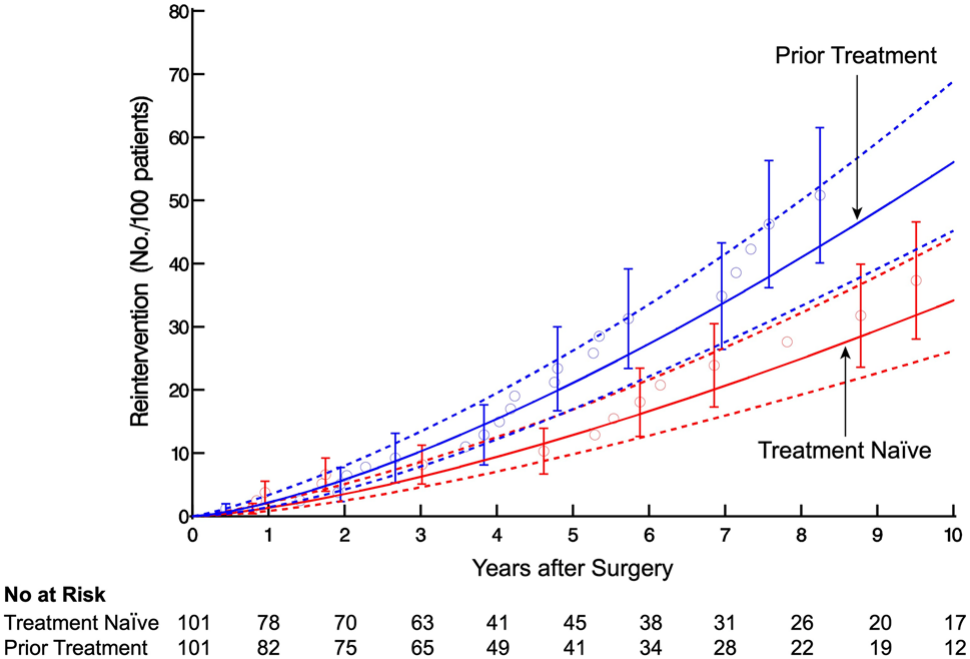
Cumulative number of reinterventions per 100 patients in the matched cohort. Blue represents patients with prior treatment and red patients who are treatment-naïve. Each circle represents a reintervention positioned by non-parametric estimate. Vertical bars represent asymmetric 68% confidence limits. Solid lines and dashes lines represent the parametric estimates and confidence intervals, respectively (Color figure online)

**Fig. 4:**
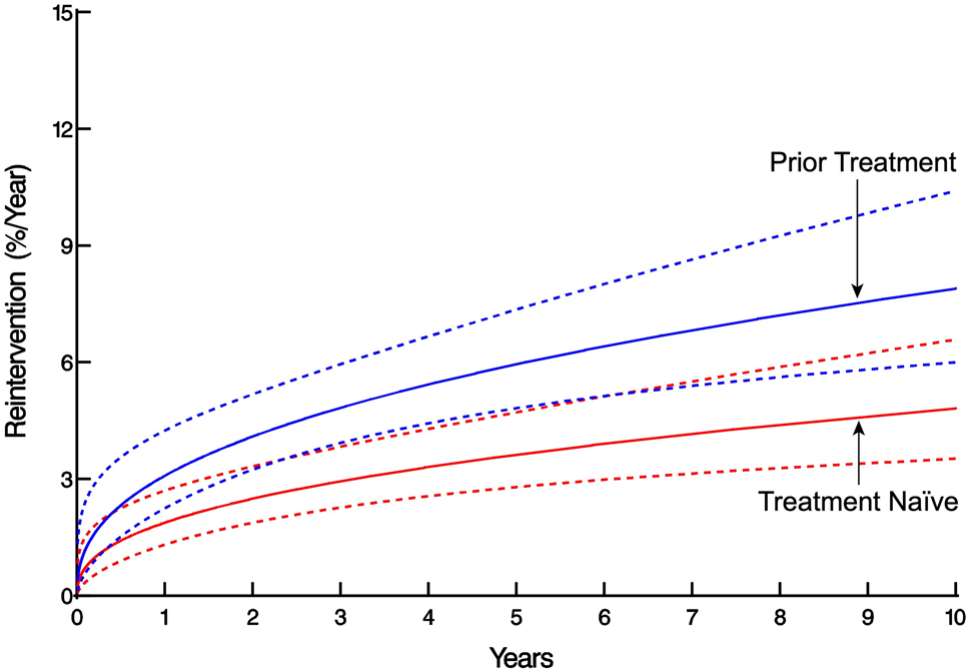
Hazard function for reintervention for matched patients with prior treatment (blue) vs patients who are treatment-naïve (red). Dashed bands represent 68% confidence intervals equivalent to 1 standard deviation (Color figure online)

Prior to propensity matching, patients in the Prior Group were slightly older, less often African American race, and more often Type I achalasia. (Supplemental Table 1, Supplemental Fig. 3). Following propensity matching, preoperative characteristics, including age, sex, race, BMI, ASA class, and achalasia subtype were similar between groups (Table 1, Supplemental Fig. 3).

#### Esophageal emptying

Due to the zero-inflated nature of postoperative TBE dimensions (with many TBEs having height and width measurements of 0 cm), we modeled the probability of complete esophageal emptying within 5 min. A multiphase non-linear logistic mixed-effects model was used to resolve several time phases to form a temporal decomposition model and to estimate the shaping parameters at each phase. PROC NLMIXED (SAS®) was used to implement the temporal decomposition model [24–26]. TBE were censored at the time of first reintervention. The group variable (with and without prior treatment) was forced into the model.

#### Symptom palliation

Longitudinal time-related analysis of Eckardt scores was performed to model the likelihood of postoperative Eckardt scores ≤ 3 using a non-linear cumulative logit mixed model (PROC NLMIXED). The prevalence of Eckardt score over time was analyzed using a non-linear cumulative logit mixed model (SAS PROC NLMIXED), with each level of the Eckardt score estimated by averaging patient specific profiles. Eckardt scores of 0, 1, 2, 3 or ≥ 4 were modeled [25]. Eckardt scores were censored at the time of first reintervention. Whether or not a patient received prior treatment was forced into the model to determine if there is a difference in the prevalence of Eckardt score over time between the two groups.

#### Reintervention

Reinterventions were treated as repeated events and defined as pneumatic dilation (≥ 30 mm), repeat Heller myotomy, per-oral endoscopic myotomy, or esophagectomy. The cumulative number of reinterventions per 100 patients across time was estimated by Nelson’s non-parametric method [27]. A parametric multiphase hazard function methodology was employed to estimate the cumulative number of events per patient [28].

## Results

### Operative details and perioperative outcomes

In the matched cohort, 99 (98%) of the Prior Group vs. 97 (97%) of the Naïve Group underwent a minimally invasive (laparoscopic or robotic) approach, with 98 (97%) and 97 (97%) receiving a Dor fundoplication (Table 2). Median operative time was 131 [103, 159] minutes in the Prior Group vs 128 [103, 161] minutes in the Naïve Group (*P* > 0.99, Table 2). Intraoperative mucosal injury occurred in no patient in the Prior Group and 3 (3%) patients in the Naïve Group, all of which were repaired laparoscopically (*P* = 0.12). Median length of stay was 1 [1, 2] day for both the Prior and Naïve groups (*P* = 0.83). One patient with prior pneumatic dilation was readmitted with a postoperative leak.

### Esophageal emptying

In the matched cohort, early postoperative TBE demonstrated complete 5-min emptying in 40 (43%) patients in the Prior Group and 39 (49%) patients in the Naïve Group (*P* = 0.49, Table 2). The predicted probability of complete 5-min emptying was 39% in the Prior Group and 50% in the Naïve Group at 1 year, decreased to 25% and 33%, respectively, at 8 years (*P* = 0.098, Fig. 1).

### Symptom palliation

Of those with available early postoperative Eckardt score, postoperative symptom palliation (Eckardt score ≤ 3) occurred in 45 (100%) patients in the Prior Group and 44 (96%) in the Naïve Group (*P* = 0.50). The longitudinal predicted probability of Eckardt score ≤ 3 was 90% for both groups at 1 year, decreasing to 81% and 83% for the Prior vs Naïve groups, respectively, at 5 years (*P* = 0.63, Fig. 2).

### Reintervention

In the matched cohort, a total of 39 reinterventions were performed in 32 patients: 24 (24%) in the Prior Group and 15 (15%) in the Naïve Group (Table 3). Overall, pneumatic dilation comprised 24 of the 39 reinterventions, followed by 10 POEM, 4 esophagectomy, and 1 redo Heller myotomy (Table 3). The cumulative number of reinterventions per 100 patients in the Prior Group compared to the Naïve Group was 21 vs 9.4 at 5 years and 56 vs 34 at 10 years, respectively, with late separation of the 68% confidence limits (*P* = 0.13, Fig. 3). The estimated risk of reintervention increased gradually for both groups over time, with slight overlap of the 68% confidence limits(*P* = 0.13, Fig. 4).

## Discussion

### Principle findings

Following Heller myotomy, we observed comparable perioperative outcomes and symptom palliation in propensity-matched patients with vs without prior pneumatic dilation and/or botulinum toxin injection. A subtle pattern demonstrating slightly increased risk of longitudinal incomplete emptying and reintervention was observed in patients with prior endoscopic interventions.

### Short-term outcomes

Endoscopic treatments may induce inflammation and consequent fibrosis at the gastroesophageal junction [3]. These changes may fuse the submucosal plane, leading to more arduous dissection. Smith et al. reported increased frequency of mucosal perforation in patients with prior treatments [2]; Portale et al. and Rakita and colleagues, on the other hand, observed a similar frequency of perforation between those with prior treatment and the treatment-naïve, more in line with our findings [5, 7]. Such variability may be explained by variable surgeon expertise, severity of fibrosis, or myotomy technique. Reassuringly, perforations were repaired minimally invasively without additional morbidity, with intraoperative mucosal injury not thought to ultimately affect postoperative outcomes [29].

### Esophageal emptying

Fibrosis from prior intervention(s) can theoretically affect postoperative emptying in two ways: it may impair intraoperative dissection, thereby affecting the *quality* of the myotomy performed, and second, it may lessen the *durability* of the myotomy, resulting in earlier recurrence of obstruction and impaired emptying. The probability of both short-term and longitudinal complete emptying was slightly lower for patients with prior endoscopic interventions; however, the gradual longitudinal decrease in complete emptying was similar for both groups. This suggests comparable *durability* of the myotomy, irrespective of prior treatment. Hence, for a small subset of patients with previous treatment, the extent of fibrosis likely affected the *quality,* or completeness, of the myotomy, resulting in the subtle, but not statistically significant, difference in emptying observed. Such an effect is to be expected, as the severity of fibrosis may vary between individual patients due to differences in the type (botulinum toxin vs pneumatic dilation) and number of interventions that patients may have received [30].

### Symptom palliation

We observed similar short-term and longitudinal symptom palliation between the previously treated and treatment-naive groups, highlighting the capability of Heller myotomy to treat patients following failed endoscopic therapy. Previous studies have described both comparable [4, 31] and suboptimal outcomes among patients with prior endoscopic treatments, although they have not utilized propensity matching to adjust for baseline patient characteristics, and few have assessed repeated longitudinal outcomes [2, 4–6, 31]. Furthermore, inability to quantify factors, such as severity of resultant fibrosis and surgeon experience, complicates comparisons between studies. While prior intervention appeared to slightly worsen longitudinal complete emptying, this did not translate to symptom palliation, in line with previous findings that complete emptying, while encouraging, is not required for symptom palliation [18, 32].

### Reintervention

Although not statistically significant, there was a pattern suggesting that patients with previous treatment may be at slightly higher risk for reintervention over time. Snyder et al. reported a similar phenomenon, with patients with > 1 preoperative intervention at greatest risk for reintervention [8]. This also supports Smith and colleagues finding, that patients with prior treatment were more likely to require redo myotomy or esophagectomy than the treatment-naïve, although neither assessed risk of reintervention over time [2]. Perhaps most importantly, our observed difference was small, with the large majority of reinterventions being endoscopic in nature. Based on this, we recommend upfront surgery when the necessary expertise is available. That said, if patients do undergo initial endoscopic treatment, they should be assured that Heller myotomy can obviate the need for further invasive surgery in most patients.

### Strengths and limitations

The strengths of our study include our repeated longitudinal assessment of both objective (emptying) and subjective (symptoms) endpoints, with use of propensity matching to account for baseline between-group differences. Additionally, the surgeries were performed by 3 surgeons within a high-volume contemporary achalasia practice that utilized predominantly minimally invasive approaches. Although the sample size of patients with prior interventions is relatively large, we were still underpowered in our ability to discriminate small between-group differences. As a result, we were not able to further stratify patients with prior interventions based on the type and/or number of intervention(s), which may impact the severity of fibrosis. Furthermore, because most preoperative interventions were performed at outside hospitals, we could not account for time between endoscopic intervention(s) and myotomy or the number of pre-myotomy inventions performed, potentially important factors.

Perhaps most importantly, patients returning for myotomy after endoscopic intervention are likely inherently “higher risk” than the treatment-naïve, as they represent a subset of patients that have already “failed” endoscopic treatment. Undoubtedly, some patients (perhaps with less severe disease) underwent pneumatic dilation or botulinum toxin injection, never required additional therapy, and were therefore not captured in the study cohort [33]. Propensity matching cannot adjust for this bias, which may be best avoided in the future with prospective studies or databases comprised of allcomers with achalasia, whether initially managed with endoscopic or surgical intervention.

### Conclusion

Heller myotomy is safe and facilitates longitudinal symptom palliation for most patients with achalasia, irrespective of prior endoscopic intervention. A subtle pattern of worse esophageal emptying and increased reintervention in patients with prior intervention suggests that when surgical expertise is available, initial management with myotomy may be preferred.

## Supplementary Information

Below is the link to the electronic supplementary material.

Supplementary file1 (JPG 165 KB)— The annual proportion of patients undergoing prior endoscopic interventions as compared to treatment naïve patients. The solid blue line represents a smoothing spline curve and the red dots the percentage of patients with prior treatment each year

Supplementary file2 (JPG 173 KB)— Completeness of follow up. Red represents subjects who underwent reintervention, green subjects deceased, and purple all remaining subjects

Supplementary file3 (JPG 514 KB)— Standardized differences of covariable balance between prior treatment and treatment naïve groups. Triangles represent before and squares after matching

Supplementary file4 (JPG 225 KB)— Distribution of propensity scores for prior treatment (red) and treatment naïve (blue) groups before and after matching. The blue and red areas represent the 101 matched pairs, and the unshaded areas the unmatched patients within the two groups

Supplementary file5 (DOCX 17 KB)

Supplementary file6 (DOCX 19 KB)

Supplementary file7 (DOCX 14 KB)
