# Supplementary figures and images for: Heller myotomy in patients with prior endoscopic interventions vs the treatment-naïve
Source: Surg Endosc. 2025 Apr 15;39(5):3328–36. doi: 10.1007/s00464-025-11661-0 (PMC12041173; doi:10.1007/s00464-025-11661-0)

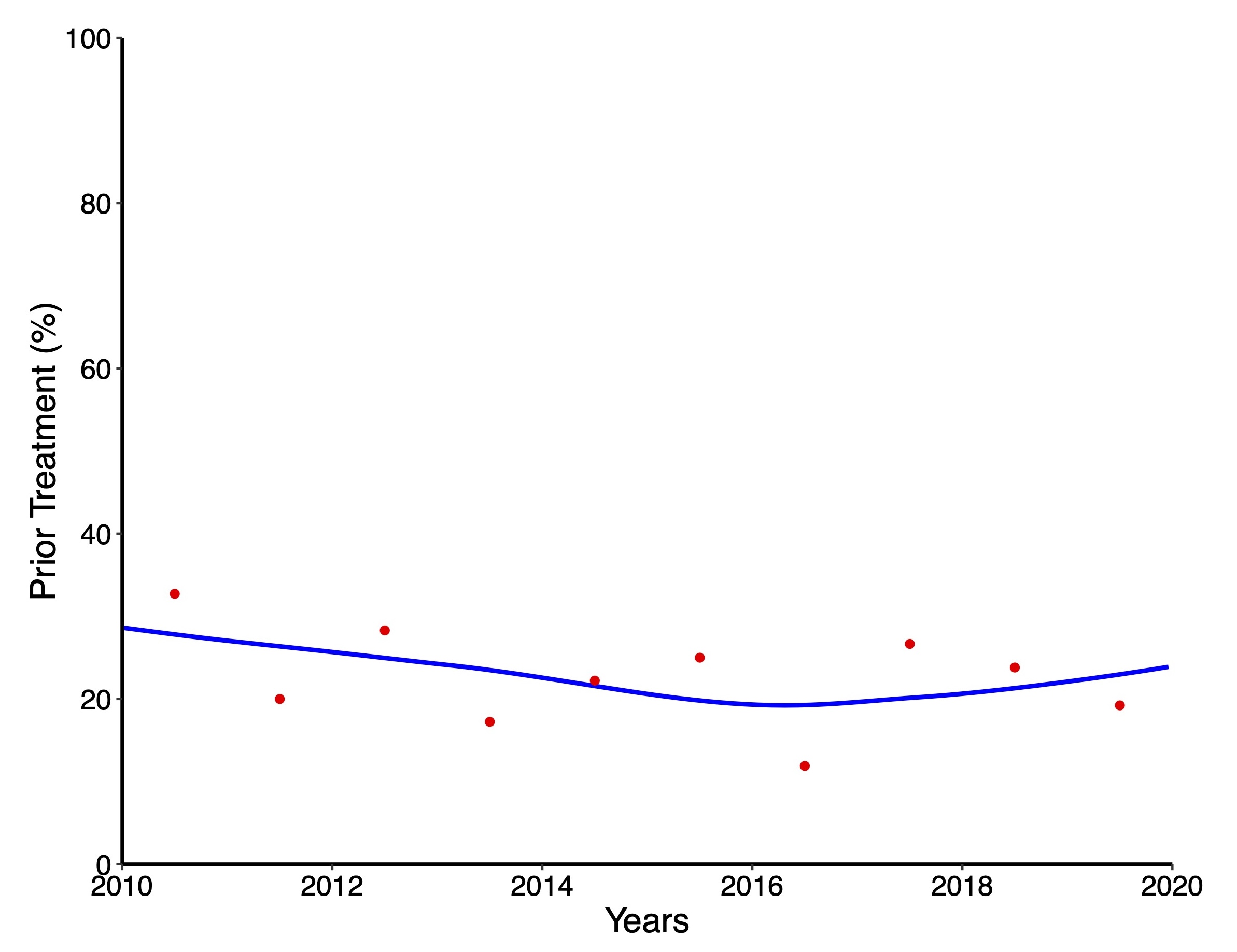

Supplement: Supplementary file 1 — Supplementary file1 (JPG 165 KB)— The annual proportion of patients undergoing prior endoscopic interventions as compared to treatment naïve patients. The solid blue line represents a smoothing spline curve and the red dots the percentage of patients with prior treatment each year [file 464_2025_11661_MOESM1_ESM.jpg]

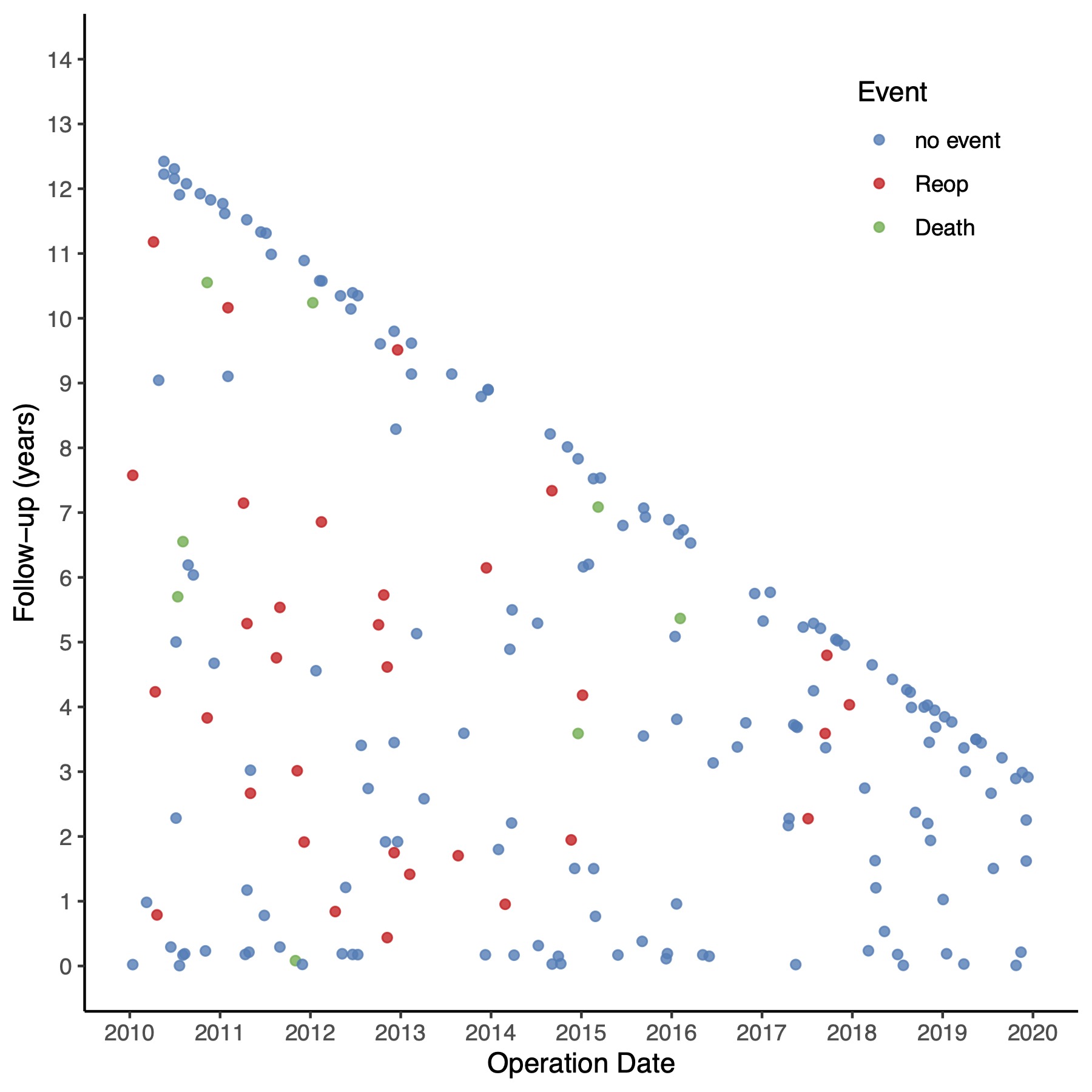

Supplement: Supplementary file 2 — Supplementary file2 (JPG 173 KB)— Completeness of follow up. Red represents subjects who underwent reintervention, green subjects deceased, and purple all remaining subjects [file 464_2025_11661_MOESM2_ESM.jpg]

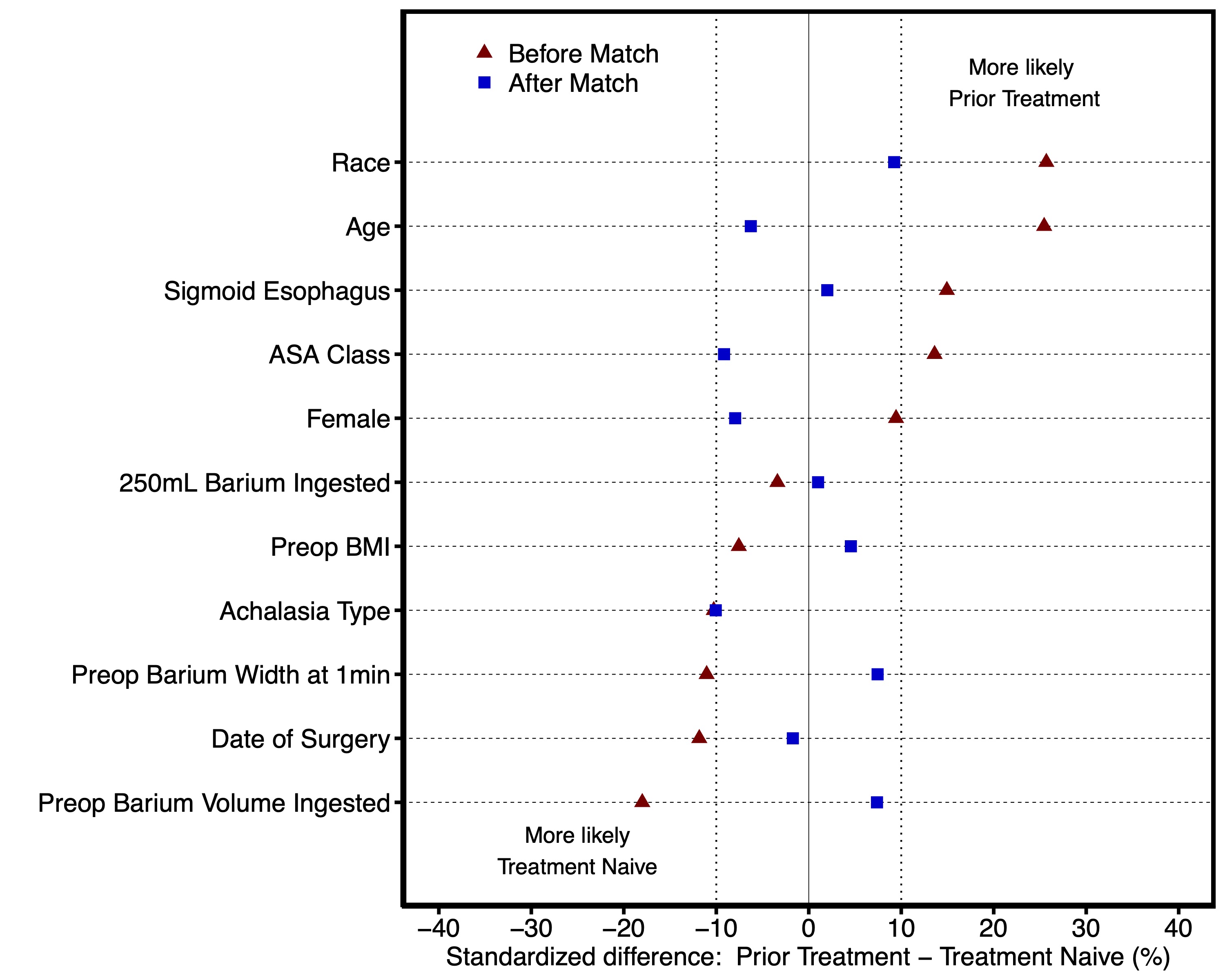

Supplement: Supplementary file 3 — Supplementary file3 (JPG 514 KB)— Standardized differences of covariable balance between prior treatment and treatment naïve groups. Triangles represent before and squares after matching [file 464_2025_11661_MOESM3_ESM.jpg]

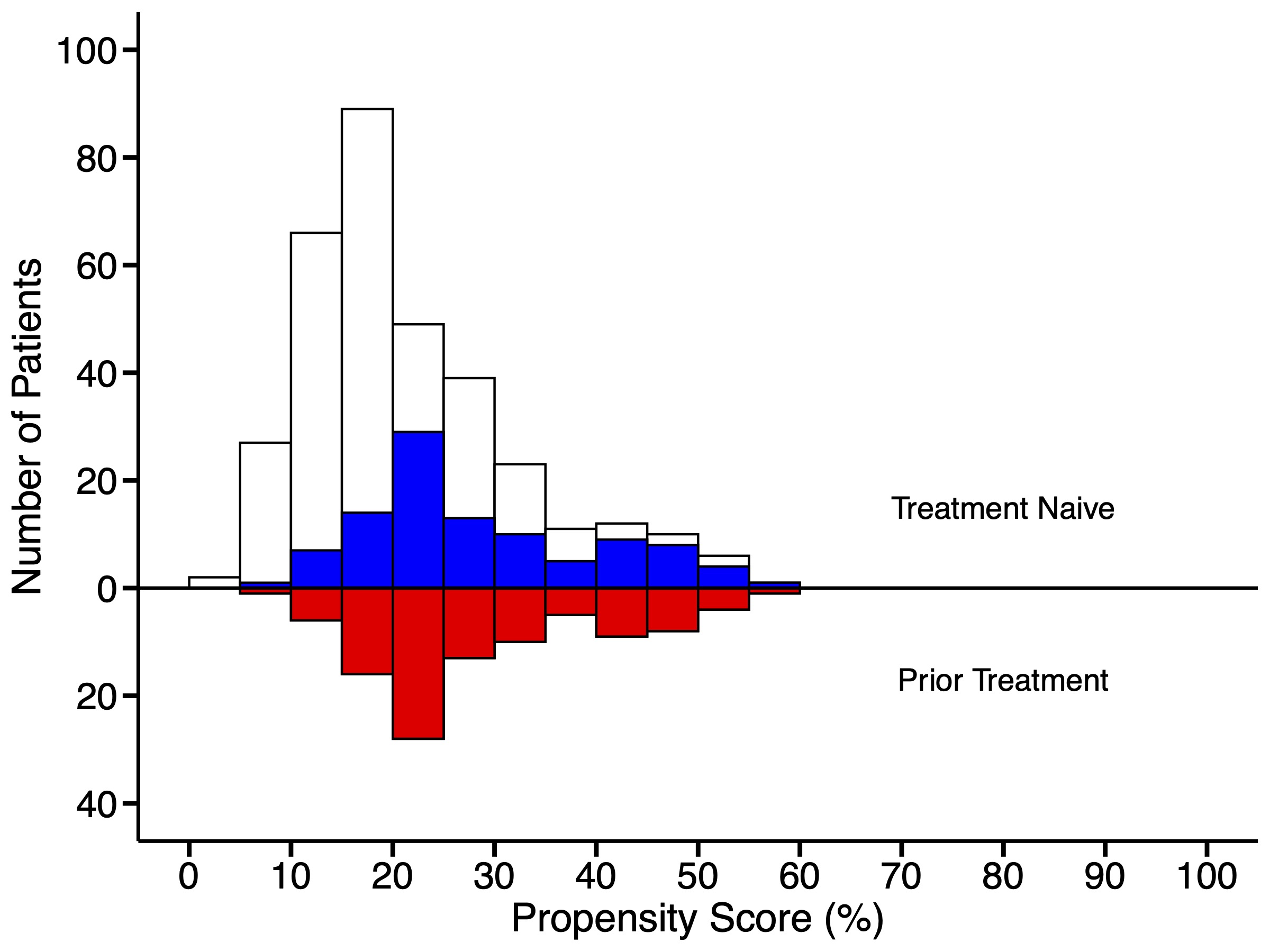

Supplement: Supplementary file 4 — Supplementary file4 (JPG 225 KB)— Distribution of propensity scores for prior treatment (red) and treatment naïve (blue) groups before and after matching. The blue and red areas represent the 101 matched pairs, and the unshaded areas the unmatched patients within the two groups [file 464_2025_11661_MOESM4_ESM.jpg]
