# Supplementary material for: Heller myotomy in patients with prior endoscopic interventions vs the treatment-naïve
Source: Surg Endosc. 2025 Apr 15;39(5):3328–36. doi: 10.1007/s00464-025-11661-0 (PMC12041173; doi:10.1007/s00464-025-11661-0)
Supplement: Supplementary file 5 — Supplementary file5 (DOCX 17 KB) [file 464_2025_11661_MOESM5_ESM.docx]

**Supplemental Table 1**. Demographic and preoperative clinical characteristics prior to propensity matching

|  | **Prior treatment**  **(N = 101)** | | **Treatment-naïve**  **(N = 335)** | |  |
| --- | --- | --- | --- | --- | --- |
| **Variable** | **Available N** | **Count(%) or Mean ± SD** | **Available N** | **Count(%) or Mean ± SD** | **SMD^c^** |
| **Age** | 101 | 52.4 ± 14 | 335 | 48.5 ± 16.5 | 25 |
| **Female sex** | 101 | 48(48) | 335 | 175(52) | 9.4 |
| **Body Mass Index** | 96 | 26.2 ± 5.5 | 326 | 26.6 ± 5.22 | -7.6 |
| **Achalasia subtype** | 80 |  | 302 |  | 24 |
| Type 1 |  | 21(26) |  | 60(20) |  |
| Type 2 |  | 53(66) |  | 224(74) |  |
| Type 3 |  | 3(3.8) |  | 9(3) |  |
| Other |  | 3(3.8) |  | 9(3) |  |
| **ASA^a^ class** | 95 |  | 326 |  | 14 |
| I |  | 1 (1.1) |  | 10 (3.1) |  |
| II |  | 27 (28) |  | 100 (31) |  |
| III |  | 51 (54) |  | 175 (54) |  |
| IV |  | 16 (17) |  | 41 (13) |  |
| **Botulinum toxin** | 101 | 62(61) | 335 | 0(0) | -- |
| **Pneumatic dilation** | 101 | 44(44) | 335 | 0(0) | -- |
| **Preop TBE^b^ sigmoid esophagus** | 96 | 21(22) | 315 | 51(16) | 15 |
| ^a^American society of anesthesiologists; ^b^Timed barium esophagram, ^c^Standardized mean difference | | | | | |
