# Supplementary material for: Heller myotomy in patients with prior endoscopic interventions vs the treatment-naïve
Source: Surg Endosc. 2025 Apr 15;39(5):3328–36. doi: 10.1007/s00464-025-11661-0 (PMC12041173; doi:10.1007/s00464-025-11661-0)
Supplement: Supplementary file 6 — Supplementary file6 (DOCX 19 KB) [file 464_2025_11661_MOESM6_ESM.docx]

**Supplemental Table 2**. Perioperative and short-term outcomes prior to propensity matching

|  | **Prior treatment**  **(N = 101)** | | **Treatment-naïve**  **(N = 335)** | |  |
| --- | --- | --- | --- | --- | --- |
| **Variable** | **Available N** | **Count(%) or 15/50/85^th^ percentiles** | **Available N** | **Count(%) or 15/50/85^th^ percentiles** | ***P*-value** |
| **Surgical Approach** | 101 |  | 334 |  | .12 |
| Robotic |  | 29 (29) |  | 131 (39) |  |
| Laparoscopic |  | 70 (69) |  | 200 (60) |  |
| Open |  | 2 (2.0) |  | 3 (0.90) |  |
| **Fundoplication type** | 101 |  | 100 |  | .21 |
| Dor |  | 98 (97) |  | 330 (99) |  |
| No fundoplication |  | 3 (3.0) |  | 4 (1.2) |  |
| **Operative duration, min** | 54 | 103/131/159 | 212 | 103/129/164 | .8 |
| **Intraoperative mucosal perforation** | 101 | 0(0) | 333 | 6(1.8) | .17 |
| **Postoperative leak** | 101 | 1(0.99) | 335 | 0(0) | .07 |
| **Length of stay, days** | 101 | 1/1/2 | 335 | 1/1/2 | .88 |
| **Symptoms^a^** |  |  |  |  |  |
| Dysphagia | 84 | 36(43) | 185 | 57(31) | .054 |
| Regurgitation | 84 | 15(18) | 185 | 18(9.7) | .06 |
| Chest Pain | 81 | 9(11) | 183 | 29(16) | .31 |
| **Total Eckardt^a^ Score** | 45 |  | 169 |  | .7 |
| 0 |  | 24 (53) |  | 102 (60) |  |
| 1 |  | 16 (36) |  | 39 (23) |  |
| 2 |  | 5 (11) |  | 22 (13) |  |
| 3 |  | 0 (0) |  | 3 (1.8) |  |
| 4 |  | 0 (0) |  | 1 (0.59) |  |
| 5 |  | 0 (0) |  | 2 (1.2) |  |
| **TBE^b^ complete emptying** |  |  |  |  |  |
| 1 minute | 91 | 14(15) | 273 | 43(16) | .93 |
| 5 minutes | 92 | 40(43) | 273 | 144(53) | .12 |
| **DeMeester score** | 56 |  | 161 |  | .18 |
| Abnormal (>14.72) |  | 11(20) |  | 20(12) |  |
| ^a^Measured within 6 months postoperatively, ^b^Timed barium esophagram | | | | | |
