# Supplementary material for: Heller myotomy in patients with prior endoscopic interventions vs the treatment-naïve
Source: Surg Endosc. 2025 Apr 15;39(5):3328–36. doi: 10.1007/s00464-025-11661-0 (PMC12041173; doi:10.1007/s00464-025-11661-0)
Supplement: Supplementary file 7 — Supplementary file7 (DOCX 14 KB) [file 464_2025_11661_MOESM7_ESM.docx]

**Supplemental Table 3**. Variables utilized for propensity analysis.

| **Category** | **Variables** |
| --- | --- |
| Demographics | Gender, age (years), body mass index, Race |
| Preoperative variables | American Society of Anesthesiologists Class, achalasia type, sigmoid morphology |
| Timed barium esophagram | Barium column widths at 1 and 5 minutes, preoperative barium volume |
| Other | date of surgery |
|  |  |
